# A phase I dose-finding, pharmacokinetics and genotyping study of olaparib and lurbinectedin in patients with advanced solid tumors

**DOI:** 10.1038/s41598-021-82671-w

**Published:** 2021-02-24

**Authors:** Andres Poveda, Ana Oaknin, Ignacio Romero, Angel Guerrero-Zotano, Lorena Fariñas-Madrid, Victor Rodriguez-Freixinos, Pedro Mallol, Raquel Lopez-Reig, Jose Antonio Lopez-Guerrero

**Affiliations:** 1 Oncogynecologic Department, Initia Oncology, Hospital Quironsalud Valencia, Avda Blasco Ibañez, 14, 46 010 Valencia, Spain; 2 Medical Oncology Department, Vall D’Hebron University Hospital, Vall D’Hebron Institute of Oncology (VHIO), Barcelona, Spain; 3 Department of Medical Oncology, Fundación Instituto Valenciano de Oncología (IVO), Valencia, Spain; 4 Department of Medical Oncology and Hematology, Odette Cancer Centre, Sunnybrook Health Sciences Centre, Toronto, Canada; 5 Clinical Trials Department, FINCIVO (Fundación de Investigación Clínica del IVO), Valencia, Spain; 6 Laboratory of Molecular Biology, Fundación Instituto Valenciano de Oncología, Valencia, Spain; 7 IVO-CIPF Joint Research Unit of Cancer, Príncipe Felipe Research Center (CIPF), Valencia, Spain; 8 Department of Pathology, School of Medicine, Catholic University of Valencia ‘San Vicente Martir’, Valencia, Spain

**Keywords:** Cancer, Molecular biology, Medical research, Oncology

## Abstract

The poly (ADP-Ribose) polymerase (PARP) inhibitor olaparib has shown antitumor activity in patients with ovarian or breast cancer with or without *BRCA1/2* mutations. Lurbinectedin is an ecteinascidin that generates DNA double-strand breaks. We hypothesized that the combination of olaparib and lurbinectedin maximizes the DNA damage increasing the efficacy. A 3 + 3 dose-escalation study examined olaparib tablets with lurbinectedin every 21 days. The purpose of this phase I study is to determine the dose-limiting toxicities (DLTs) of the combination, to investigate the maximum tolerated dose (MTD), the recommended phase II dose (RP2D), efficacy, pharmacokinetics, in addition to genotyping and translational studies. In total, 20 patients with ovarian and endometrial cancers were included. The most common adverse events were asthenia, nausea, vomiting, constipation, abdominal pain, neutropenia, anemia. DLT grade 4 neutropenia was observed in two patients in dose level (DL) 5, DL4 was defined as the MTD, and the RP2D was lurbinectedin 1.5 mg/m^2^ + olaparib 250 mg twice a day (BID). Mutational analysis revealed a median of 2 mutations/case, 53% of patients with mutations in the homologous recombination (HR) pathway. None of the patients reached a complete or partial response; however, 60% of stable disease was achieved. In conclusion, olaparib in combination with lurbinectedin was well tolerated with a disease control rate of 60%. These results deserve further evaluation of the combination in a phase II trial.

## Introduction

Olaparib is a potent oral poly (ADP-Ribose) polymerase (PARP) inhibitor that leads to synthetic lethality in patients with BRCA1/2 deficient tumor cells^1^. Moreover, increasing data suggest that PARP inhibitors may have a role in the treatment of sporadic high-grade serous ovarian and other cancers with homologous recombination deficiency (HRD)^2^. Continuous oral olaparib showed efficacy in heavily pretreated mutated BCRA1/2 breast cancer^3–5^. Olaparib as monotherapy improved progression-free survival (PFS) in patients with platinum-sensitive relapsed serous ovarian cancers regardless of *BRCA* status^6–9^, and in *BRCA* mutated patients with ovarian and breast cancers previously treated with chemotherapy^10^.

In addition to the single-agent studies, olaparib has been combined with chemotherapy since it might act as sensitizer by limiting DNA damage repair^11^. Phase I and II clinical trials assessing olaparib combined with standard chemotherapy have shown encouraging results in ovarian and breast cancers^12–16^.

Lurbinectedin (PM01183) is a new synthetic alkaloid, structurally related to ecteinascidins. In common with trabectedin, lurbinectedin contains a pentacyclic skeleton composed of two fused tetrahydroisoquinoline rings (subunits A and B) that is mostly responsible for DNA recognition and binding. The additional module (ring C) in lurbinectedin is a tetrahydro β-carboline rather than the additional tetrahydroisoquinoline present in trabectedin. This structural difference may confer pharmacokinetic benefits as well as intrinsic activity^17^. Lurbinectedin inhibits the transcription process through its binding to CG-rich sequences, induces the generation of double-strand DNA breaks and subsequent apoptosis^18^ and also reduces tumor-associated macrophages and the inflammatory microenvironment through the inhibition of inflammatory factors^19^. Since these DNA double-stand breaks are processed through homologous recombinant repair (HRR), trabectedin and lurbinectedin are particularly associated with sensitivity in HRR-deficient cells^20^. A recent study reported an impressive activity in platinum-resistant ovarian cancer patients in terms of ORR, PFS and OS^21^.

The combination of olaparib, an inhibitor of DNA damage repair (DDR), with a DNA damaging agent such as lurbinectedin seems an interesting approach to maximize the effect of DNA damage. In preclinical models, the combination of both agents was synergistic and led to biologically significant deregulation of the DDR machinery that elicited relevant antitumor activity^22^. However, the major concern over the combinations is overlapping toxicities. Single-agent lurbinectedin showed significant hematological toxicity with grade 3–4 neutropenia up to 85% when administered at flat dose^21^, being lower (57%) when the dose is adjusted to body surface area^23^. Moreover, treatment with chemotherapy and continuous dosing of olaparib is usually not feasible due to the high rate of hematologic adverse events. An intermittent schedule of olaparib is better tolerated than a continuous one when combined with chemotherapy^14,24^.

This phase I dose-escalation trial was designed to determine the safety and the recommended dose of lurbinectedin combined with a short course of olaparib administered every three weeks in patients with advanced solid tumors.

## Results

### Patient characteristics and treatment

**Figure 1:**
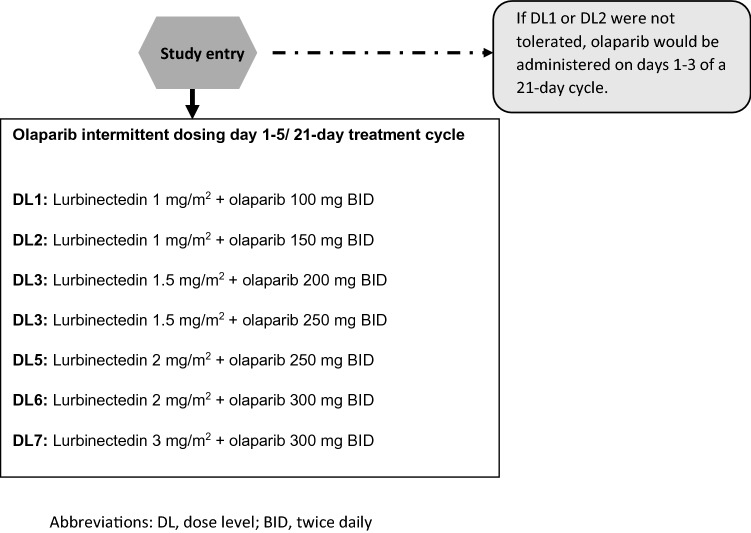
The 3 + 3 dose-escalation study design.

**Figure 2:**
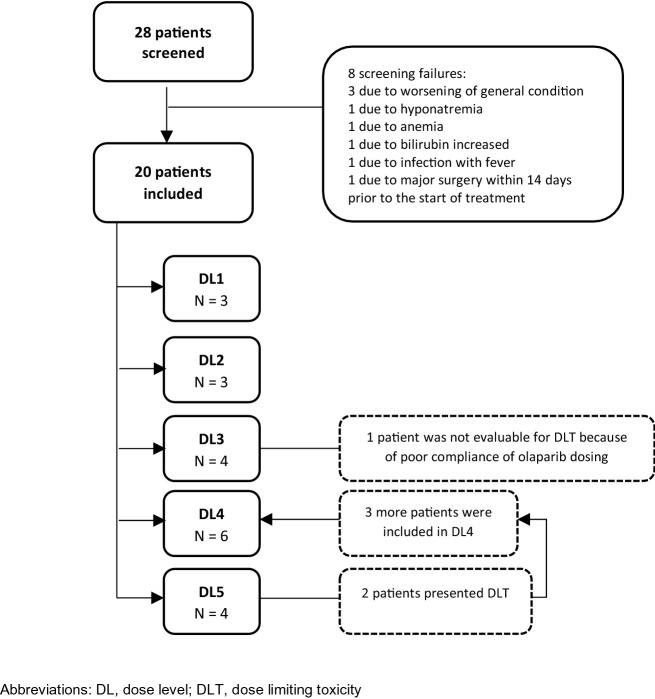
Consort diagram.

Figure 1 depicts the 3 + 3 dose-escalation study design; the Consort diagram is in Fig. 2. A total of 20 patients were enrolled in this phase I trial in five dose levels (DLs) (Table 1): 3 in DL1 and DL2, 4 in DL3 and DL5, and 6 in DL4. Since no dose-limiting toxicity was observed in DL1 and DL2, with olaparib administered from day 1 to 5, the study continued with the same 5-day schedule of oral olaparib from DL3 and beyond. The safety and efficacy analyses included all 20 patients. Most of the patients (75%) in the trial finished study treatment due to radiological progression, while 25% ended because of clinical progression (one patient in DL1 and one patient in DL5), toxicity (asthenia grade 3 in one patient in DL3 and hematological toxicity in one patient in DL5) and patient decision (one patient in DL3). Table 1 shows the patient characteristics for each cohort. All patients were female, and the most common type of tumour was ovarian cancer (n = 15; 75%). Median time from the primary tumour diagnosis and the inclusion in the trial was 33.9 (range 1.8–108.4) for DL5 and 80.3 months (range 10.9–127.1) for DL1. All patients included in this trial had previously been treated with systemic chemotherapy, with a median of 4–5 lines (range 1–7). Only one patient at DL1 had received 1 line and was platinum resistant.

**Table 1:** Patient characteristics.

Characteristic	DL1 n = 3	DL2 n = 3	DL3 n = 4	DL4 n = 6	DL5 n = 4
Age, median (range) (years)	62 (51–69)	60 (51–63)	62.5 (52–69)	56.5 (54–60)	61 (57–67)
**ECOG PS, n (%)**					
0	2 (66.7)	2 (66.7)	1 (25)	5 (83.3)	2 (50)
1	1 (33.3)	1 (33.3)	3 (75)	1 (16.7)	2 (50)
BSA, median (range) (m^2^)	1.59 (1.57–1.65)	1.9 (1.63–2.03)	1.78 (1.69–1.92)	1.78 (1.63–1.82)	1.59 (1.54–1.63)
**Primary tumor type, n (%)**					
Ovarian cancer	3 (100)	3 (100)	2 (50)	5 (83.3)	2 (50)
High histological grade	3 (100)	2 (66.7)	2 (100)	5 (100)	1 (25)
Endometrium cancer	–	–	2	–	1 (25)
Uterine leiomyosarcoma	–	–	–	1 (17.7)	
Time from diagnosis to the inclusion, median (range) (months)	80.3 (11–127)	38.1 (26–61)	71.5 (58–99)	56.1 (39–61)	33.9 (16–72)
Lines of previous systemic therapy, median (range)	5 (1–6)	4 (4–6)	4 (2–6)	5 (3–6)	5 (3–7)
Previous radiotherapy, n (%)	–	–	2 (50)		1 (25)
**Metastasis at baseline, n (%)**					
Lung	–	–	1 (25)	1 (16.7)	2 (50)
Liver	1 (33.3)	1 (33.3)	1 (25)	1 (16.7)	2 (50)
Lymph nodes	3 (100)	1 (33.3)	2 (50)	3 (50)	2 (50)
Bone	–	1 (33.3)	–	–	–
Others	–	1 (33.3)	1 (25)	3 (50)	2 (50)
Treatment duration, median (range) (days)	45 (25–132)	27 (26–27)	127 (48–270)	87 (26–440	104 (59–180)
Number of cycles of olaparib, median (range)	2 (2–7)	2 (2–2)	6.5 (3–12)	5 (2–19)	4.5 (2–8)
Number of cycles of lurbinectedin, median (range)	3 (2–7)	2 (2–2)	6.5 (3–12)	5 (2–19)	4.5 (2–9)
Total cumulative dose of olaparib, median (range) (mg)	2000 (1700–6900)	3000 (3000–3000)	13,000 (5600–24,000)	12,500 (5000–15,000)	10,500 (6750–14,500)
Total cumulative dose of lurbinectedin, median (range) (mg)	5.1 (3.2–11.2)	3.9 (3.4–4)	17.5 (8.4–32.1)	12.4 (5.7–41.5)	12.3 (6.2–20)

*BSA* body surface area, *DL* dose level, *ECOG PS* Eastern Cooperative Oncology Group Performance Status.

### Exposure to treatment

The median study treatment duration was from 27 days for DL2 to 127 days for DL3 (Table 1), while the maximum median number of cycles of olaparib and lurbinectedin was 6.5 for DL3. Patients from DL1 and DL2 received the fewest median number of cycles of lurbinectedin and olaparib.

The median cumulative dose of olaparib was higher at each subsequent cohort until DL3 (2000, 3000 and 13,000 mg). Nevertheless, the median cumulative dose of olaparib in DL4 (13,500 mg) and DL5 (10,500 mg) was lower than in DL3 because of the more frequent dose reductions related to toxicity secondary to higher olaparib dose and the lower number of cycles administered in these DLs (median number of cycles were 6.5, 5 and 4.5 for DL3, DL4 and DL5, respectively).

Regarding lurbinectedin, the highest cumulative dose of lurbinectedin was administered in patients in DL3 because of the increased number of cycles administered and the lower rate of dose modifications. Most dose modifications (n = 3, 75%) occurred in the DL5 and were related to hematological toxicity.

### Safety

All patients experienced ≥ 1 AE (Table 2). A total of 280 AEs were reported, being 167 events considered by the investigator related to study drugs. AEs (mostly grade 1–2) observed in ≥ 10% of all patients and by dose level are shown in Supplementary Table 1. Most common adverse events were: asthenia (55%), nausea (55%), vomiting (50%), constipation (45%), abdominal pain (40%), neutropenia (35%) and anemia (35%). Incidence of grade 3–4 AEs was low and limited to DL3-DL5. Asthenia (15%) is the unique grade 3 non-hematological toxicity observed, whilst hematological toxicity was grade 3 anemia in two patients (10%) and grade 3 and 4 neutropenia in 1 (5%) and two patients (10%), respectively. One patient at DL3 and another one at DL5 discontinued the treatment because of AEs.

**Table 2:** Overview of safety.

Total of patients with ≥ AEs, n (%)	DL1 n = 3	DL2 n = 3	DL3 n = 4	DL4 n = 6	DL5 n = 4
Any AEs	3 (100)	3 (100)	3 (100)	6 (100)	4 (100)
Serious AE	–	–	1 (25)	–	2 (50)
Grade 3–4 AE			3 (75)	–	1 (25)
Treatment-related AE	3 (100)	2 (66.7)	4 (100)	4 (66.7)	4 (100)
Treatment-related serious AE	–	–		–	1 (25)
Treatment-related Grade 3–4 AE	3 (100)	2 (66.7)	4 (100)	4 (66.7)	4 (100)
AE leading to treatment discontinuation			1 (25)		1 (25)
AE leading to olaparib dose delay or modification			2 (50)	1 (16.6)	2 (50)
AE leading to lurbinectedin dose delay or modification			2 (50)	1 (16.6)	2 (50)
AE leading to DLT	–	–	–	–	2 (50)

*AE* adverse event, *DL* dose level, *DLT* dose limiting toxicity.

DLTs were observed in two patients who presented grade 4 neutropenia related to the combination of lurbinectedin and olaparib in DL5. Therefore, DL4 was defined as the MTD, and the RP2D was lurbinectedin 1.5 mg/m^2^ + olaparib 250 mg BID. No deaths related to the treatment occurred.

### Pharmacokinetics

Pharmacokinetic data were available in 19 patients for lurbinectedin and olaparib. For the three lurbinectedin DLs explored, the mean lurbinectedin clearance (CL) was close to 12 L/h (Supplementary Fig. 1). Median lurbinectedin CL presented large variability (92%) at dose of 1 mg/m^2^ because one patient had high CL (37 L/h), while for the 1.5 mg/m^2^ and 2 mg/m^2^ DLs the variabilities were 36% and 52%, respectively (Supplementary Table 2). The stratification of lurbinectedin PK parameters by olaparib DL showed a large CL variability when lurbinectedin was administered at 1 mg/m^2^ (olaparib 100 and 150 mg BID). In comparison, the next olaparib DLs (200 and 250 mg BID) matched with lurbinectedin DLs of 1.5 and 2 mg/m^2^, the CL variabilities were close to 36% (Supplementary Fig. 2 and Table 3). At the highest olaparib DL (250 mg BID), mean lurbinectedin CL was 12 L/h which is the same to the mean CL as single-agent^25^ (Supplementary Fig. 2 and Table 3). We did not observe any potential drug–drug interactions (DDI) for lurbinectedin.

Regarding olaparib, PK parameters were calculated using noncompartmental analysis (NCA), and these did not show differences by lurbinectedin DL. When the parameters were stratified by olaparib DL, the CL at 100 mg was 3.0 L/h and higher (6–10 L/h) for the next DLs (Supplementary Fig. 3 and Table 3).

### Efficacy

All 20 patients were evaluable for efficacy. The best global response was stable disease in 12 (60%) patients. By cohort, 66.67% in DL1, 75% in DL3, 66.67% in DL4 and 75% of patients in DL5 achieved stabilization. None of the patients reached a complete or partial response in this trial.

An exploratory analysis of time to radiological progression (TTRP) was performed for the global population (Supplementary Fig. 4) and by DL (Supplementary Fig. 5). Median TTRP was 4.0 months (CI 95% 1.8–6.1) for the global population (Supplementary Fig. 4). TTRP could not be analyzed in patients from DL1 since two out of three patients were censored due to clinical progression. For the other DLs (2–5), TTRP was 1.8, 6.0, 3.6 and 6.1 months, respectively (Supplementary Fig. 5).

### Translational studies

### Chemosensitivity to lurbinectedin and olaparib monotherapy and combined treatment

Drug sensitivity was evaluated after fixing doses and time exposure to the drug. The dose–response curves were performed in the complete panel of ovarian cancer cell lines for lurbinectedin and olaparib. Except for OVCAR3, all cell lines presented a great sensitivity to lurbinectedin, showing an IC50 below 1 nM (Supplementary Fig. 6). However, olaparib's sensitivity range was found at a micromolar scale, thus facilitating the experimental and analytical procedures. In this case, cell lines showed higher differences in IC50, varying from 2.9 to > 100 µM (Supplementary Fig. 7). IC50 comparison between HRD and no HRD cell lines showed a higher value in those not carrying mutation in this pathway, without statistical difference (Supplementary Fig. 8).

Administration of lurbinectedin and olaparib to TOV112 cell line was performed to corroborate if the drug combination was suitable for clinical application as already described^22^. These experiments confirmed the presence of significant synergism, with a peak in the dose regions of 33–15% of cell survival (CI 0.1–0.17). The most synergistic doses were between 2 and 0.5 nM for lurbinectedin and IC50 nM for olaparib (Supplementary Fig. 9).

### Western blot analysis

**Figure 3:**
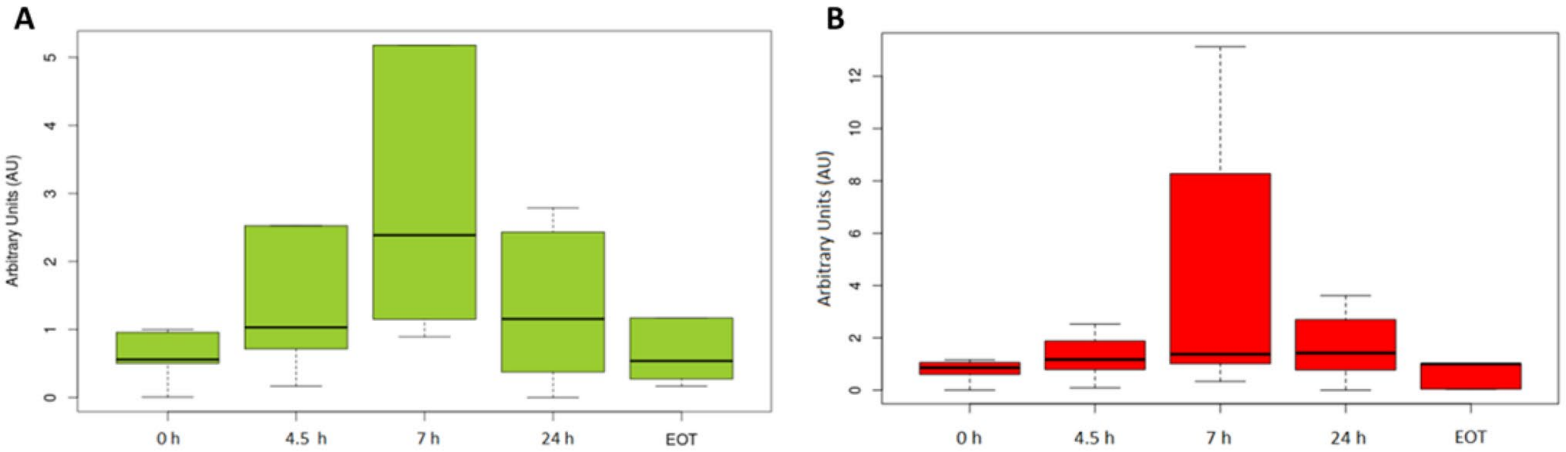
(**A**) Boxplot comparing RAD51 protein level in peripheral blood mononuclear cell (PBMC) samples at different times during treatment. (**B**) Boxplot comparing γH2AX protein’s level in PBMC samples at different times during treatment.

Protein levels of γH2AX and RAD51 were analyzed at four different time points (baseline and 4.5, 7 and 24 h after treatment administration) to determine when the changes in protein expression were statistically significant. Both proteins increased their concentration from baseline to 7 h, reaching the highest expression level and decreasing from this point forward. Only RAD51 suffered a significant protein increase (p = 0.007592) at 7 h (Fig. 3A). γH2AX levels show a trend in this sense (Fig. 3B).

### Mutational analysis results: HR stratification

**Figure 4:**
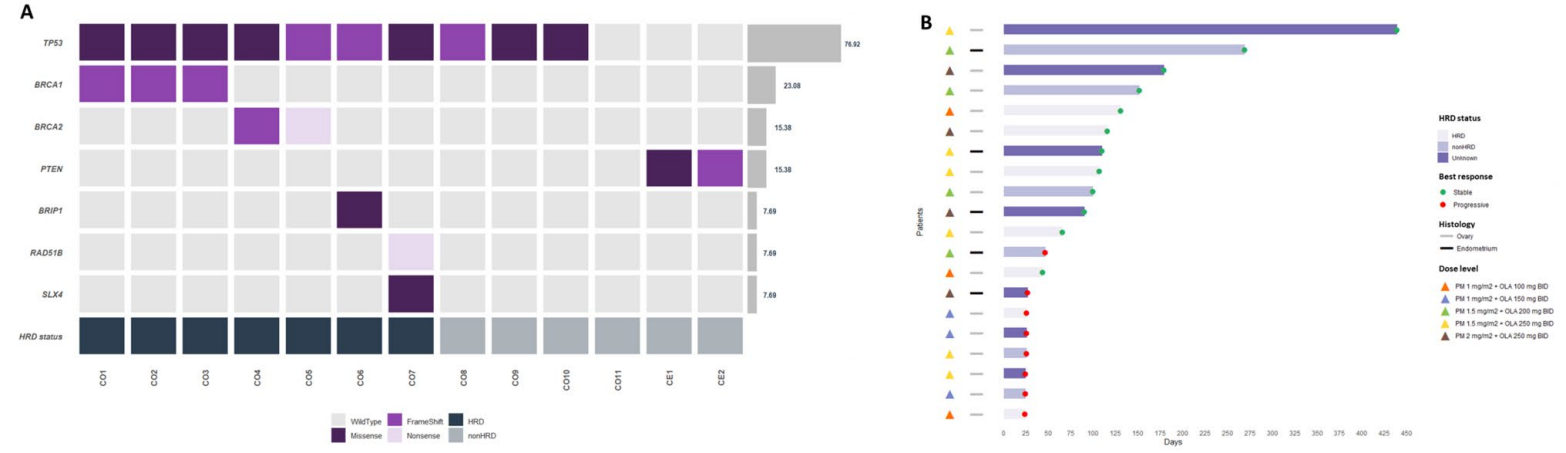
(**A**) Frequency of alterations across the studied DNA repair genes. (**B**) Main prognostic, histologic and molecular features of tumors included in the trial. *CO and CE correspond to histology type (Ovarian and endometrium, respectively).

Material for mutational analysis was available for 13 patients (11 ovarian cancer and 2 endometrial cancer patients). Among the 35 genes analyzed, 7 were found to be mutated: *BRCA1, BRCA2, BRIP1, PTEN, RAD51B, SLX4* and *TP53* (Fig. 4A). A total of 20 mutations were identified with 2 mutations/case (range 0–2). Only one sample of the series did not harbor any mutations.

*TP53* alterations were found in 76.9% (10/13) of patients. However, only 2/13 (15.4%) presented *PTEN* mutation, matching with endometrial cancer patients. Samples were classified as HR proficient (do not harbor mutation in any HR gene) or HR deficient (*BRCA1/2* or any HR gene mutated). A total of 7/13 (54%) patients presented mutations in HR pathway (Fig. 4B), five of them corresponding to *BRCA1/2* genes*.* Histology, HRD status and response of each patient was displayed in Supplementary Table 4.

## Discussion

The combination of lurbinectedin and olaparib showed a synergistic effect on cell lines that may be the reason for the increased efficacy and the toxicity observed in trials. With that in mind, we designed a dose-escalation study with different dose of lurbinectedin on day 1 and olaparib on days 1–5, but considering the possibility of reducing olaparib course to 3 days (days 1–3) as was amended to do in a previous study^13^. However, olaparib reduction was not needed since no toxicity was observed in DL1 and DL2.

To our knowledge, this is the first study where the combination of lurbinectedin and a PARP inhibitor deemed feasible. In our trial, the combination of chemotherapy with olaparib's dose schedule from day 1 to 5 was feasible. Previous combination trials using continuous olaparib had chemotherapy such as cisplatin and gemcitabine^26^, cisplatin alone^13^, and recently with carboplatin and/or paclitaxel^16^ and gemcitabine^27^ had an early stop due to high hematological toxicity. Only a continuous olaparib schedule and liposomal doxorubicin were successfully combined in a phase I study^28^ and is currently being evaluated in a phase 2 trial (NCT03161132). Hence, only administration of low dose of continuous olaparib was tolerable. However, an intermittent dosage of olaparib, especially short course, was feasible (Table 3). Combinations with intermittent olaparib capsules 200 mg BID on days 1–10 of every 21-day cycle with low dose carboplatin AUC of 4 and paclitaxel showed 43% grade 3 neutropenia^14^. Another report of intermittent olaparib and carboplatin plus paclitaxel failed to establish the MTD because more than half of the patients had dose modifications and delays due to myelosuppression^24^. Although myelosuppression is a “class-toxicity” for all PARP inhibitors, other PARP inhibitors such as veliparib have been associated with lower myelosuppression in monotherapy studies and continuous administration of veliparib has been successfully combined with chemotherapy^29,30^.

**Table 3:** Summary of main characteristics of combined phase I–II trials of olaparib and chemotherapy.

Study	Chemotherapy	iPARP dosage	PK	Toxicity	Efficacy RR/PFS	Comments
Oza (2015)^14^	CBDCA AUC4 Paclitaxel 175	Ola caps 200 mg BID day 1–10	N	Nausea 69% Neutropenia 49%	RR 64% PFS 12.2mo	Feasible, toxic, only phase 2
Van der Noll (2019)^24^	CBDCA Paclitaxel	Ola caps and tablet int	Y	Neutropenia 47%	RR 46%	Feasible,
Van der Noll (2019)^16^	CBDCA Paclitaxel	Ola caps continuous	Y	Neutropenia 51% Thrombocytopenia 25%	–	Stopped
Rajan (2012)^26^	CDDP Gemcitabine	Ola caps cont	Y	Neutropenia thrombocytopenia	–	stopped
Balmaña (2014)^13^	CDDP	Ola capsule cont	Y	Neutropenia 41% Nausea 78%	RR 41% RR 43% OC and BRCA mut	stopped
Lampert (2019)^15^	CBDCA	Ola 1–7	Y	Neutropenia 23%	RR 20% CBR 64%	feasible
Del Conte (2014)^28^	PLD	Ola tablet cont/ int	Y	Stomatitis 73% Neutropenia 30%	RR 33%	Feasible
Bendell (2015)^27^	Gemcitabine	Ola caps cont/int	Y	Hematological 55%	–	Feasible with very low doses
Present trial	Lurbinectedin	Ola tablet 1–5	Y	Asthenia 55% Neutropenia G3 15% and anemia G3 10%	RR 0% SD 60%	Feasible

*AUC* area under the curve, *CBDCA* carboplatin, *CBR* clinical benefit rate, *CDDP* cisplatin, *Cont* continuously, *G3* grade 3, *int* intermittently, *iPARP* poly (ADP-Ribose) polymerase inhibitor, *OC* ovarian cancer, *Ola* olaparib, *PFS* progression-free survival, *PLD* pegilated liposomal doxorubicin, *PK* pharmacokinetics, *RR* response rate, *SD* stable disease.

Our trial's main non-hematological toxicities were nausea and asthenia, similar to what has been reported by van der Noll in the intermittent trial^24^ and by Balmaña^13^. Neutropenia and anemia were observed in 15% and 10% of patients, respectively. The frequency of these hematological toxicities in our trial was lower than the reported previously^24^. Overall, the combination of a short course of oral olaparib and lurbinectedin was well tolerated with manageable AEs. Based on the safety results, the recommended dose for the phase II study was lurbinectedin 1.5 mg/m^2^ on day 1 and olaparib capsules 250 mg BID on days 1–5 of a 21-day cycle.

In our trial, the pharmacokinetic analysis revealed no drug interactions for lurbinectedin or olaparib. The mean olaparib total body clearance of 7 L/h in our trial was comparable with the literature, with values of 5.1 L/h and 8.6 L/h^31^. However, the terminal half-life in our trial was shorter (3 h) than previously reported (between 7 and 12 h), probably because these values were obtained from single-dose trials; while in this trial the NCA used samples until 6 h.

We did observe that both protein levels of RAD51 and H2AX increased consistently from baseline to 7 h. Regarding the limitations of the pharmacodynamic study of RAD51 and H2AX, we found pre-analytical and technical difficulties due to sample preservation and its use on western blot analysis. Massive contamination of erythrocytes in lymphocyte pellet (hemolyzed samples) made accurate colorimetric protein quantification impossible. For this reason, results from a high number of samples were unsuitable, thus being excluded from data processing.

Oza et al.^14^ conducted a randomized phase II trial to assess olaparib capsules' efficacy and tolerability plus carboplatin and paclitaxel versus carboplatin plus paclitaxel alone in patients with platinum-sensitive, recurrent, high-grade ovarian cancer. No differences in terms of response were observed between chemotherapy alone (58%) and the combination of chemotherapy plus olaparib (64%). On the other hand, the response rate of the study by van der Noll in a heavily pretreated, although highly selected ovarian cancer population, suggests a potential benefit of chemotherapy plus olaparib in a more advanced disease^24^. In our study, the combination of lurbinectedin plus olaparib did not render any response in this population of heavily pretreated epithelial ovarian and endometrial cancer without previous treatment with PARP inhibitors. However, the response rate was a secondary endpoint of the phase I trial and measurable disease was not required as an eligibility criterion. In the phase II trial we are going to assess the efficacy of the combination of olaparib and lurbinectedin in terms of response rate as the primary endpoint.

Homologous recombination deficiency (HRD) was found in the 54% of the analyzed samples, similar to previously reported data^32^. Due to the short number of available samples, the lack of responses and the trial design (dose escalation); it is not possible to identify any population that might significantly benefit from the combination.

In summary, the combination of lurbinectedin and olaparib demonstrated a safe and tolerable profile. The RP2D was lurbinectedin 1.5 mg/m^2^ on day 1 and olaparib capsules 250 mg BID on days 1–5 of a 21-day cycle. The disease control rate that we obtained in this dose-escalation trial was stable disease (60%), but further evaluation in a phase II study in gynecological tumors regardless of BRCA status is needed. To the best of our knowledge, this is the first study of olaparib-based combination with acceptable AEs. As previously mentioned, our trial has several limitations, mainly the limited number and quality of the samples to perform the translational studies.

## Material and methods

### Study design and participants

This open-label, multicenter, phase Ib, dose-escalation trial enrolled patients with advanced solid tumors without established standard therapeutic alternatives. The primary objective was to evaluate safety and toxicity, identify the dose-limiting toxicity (DLT), estimate the maximum tolerated dose (MTD) and the recommended phase II dose (RP2D) of olaparib in combination with lurbinectedin. The secondary endpoints were overall response rate (ORR), the pharmacokinetics of the combination and additional translational research focused on the study of alterations in the DNA repair machinery by HR.

The study (NCT02684318, EudraCT 2015-001141-08, 03.10.2015) was approved by the ethics committee of the Hospital Vall d’Hebron and was conducted in accordance with the Declaration of Helsinki, ICH Good Clinical Practice guidelines and the current legislation. Written informed consent was obtained from all patients before study-specific procedures.

Patients histologically confirmed advanced or metastatic solid tumors were eligible if they were 18 years of age or older, and had an Eastern Cooperative Oncology Group performance status (ECOG-PS) of 0 or 1. Other eligibility criteria included: no established effective therapy, adequate organ and bone marrow function, and ability to swallow oral medication.

### Study treatment

Patients received lurbinectedin intravenously on day 1 at a starting dose of 1 mg/m^2^ in combination with olaparib oral twice daily (BID) at an initial dose of 100 mg/12 h on days 1–5 of a cycle of 21 days. Dose escalation following a standard 3 + 3 design. Patients were enrolled in successive dose-escalations dose levels (DL) for the combination according to Fig. 1. If DL1 or DL2 were not tolerated, then the same doses of both drugs were tested but administering olaparib on days 1–3 of each cycle of 21 days. Dose-limiting toxicities (DLTs) were evaluated during cycle 1 and are in the “Supplementary Material”. MTD/RP2D was defined as the highest dose at which < 1 of 6 patients experience DLT during the first 21-day treatment period, with the next higher dose having at least two of the up to six patients undergoing a DLT during cycle 1. Intrapatient escalation was not allowed.

### Study assessments

Hematology and biochemistry tests were performed at baseline, weekly during cycle 1, and on day 1 and 15 during subsequent cycles. Electrocardiogram was done at baseline and repeated if clinically indicated.

Tumor assessment was performed according to Response Evaluation Criteria in Solid Tumors version 1.1 (RECIST v1.1) every other cycle until disease progression. ORR was defined as the proportion of patients with complete (CR) or partial response (PR). Time to radiological progression (TTRP) was defined as the time from inclusion until objective (RECIST v1.1) tumor progression. Adverse events (AE) and laboratory abnormalities were graded with the National Cancer Institute Common Terminology Criteria for Adverse Events (NCI-CTCAE) v.4.0.3 and coded using the Medical Dictionary for Regulatory Activities (MedDRA) v.14.1.

Blood samples for pharmacokinetic (PK) analyses were collected during cycle 1, with a schedule of ten samples (Supplementary Table 5).

### Translational studies

#### Cell lines and culturing

The following Ovarian Cancer Cell lines were analyzed: TOV112, SKOV-3, OVCAR-3, PEO1, PEO6, PEO4, A2780 and A2780-Cis (an A2780 variant made resistant to platinum), representing the main different molecular subtypes of ovarian cancer (Supplementary Table 6). These cell lines were maintained at 37 °C in 5% CO2 in RPMI‐1640 medium (Sigma‐Aldrich Co.) and DMEM medium (Sigma‐Aldrich Co.), all supplemented with 10% heat‐inactivated FBS (Sigma‐Aldrich Co.). To verify the genetic profile of the cell lines, a characterization of microsatellite markers was carried out (AmpFlSTR Identifiler PCR Amplification Kit, AppliedBiosystem).

#### Cytotoxicity assay

For the pharmacological characterization of the different cell lines, dose–response curves were obtained for lurbinectedin (range between 25 and 0.0343 μM and 100–1.5625 μM dilution factor 3 and 2, respectively) and olaparib (range between 10 and 0.15625 nM). The MTT (3-(4,5-dimethylthiazol-2-yl)-2,5-diphenyltetrazolium bromide) colorimetric assay measures the metabolic activity of viable cells, was used to determine the chemosensitivity of the cell lines. Cytotoxicity assay was assessed performing six technical replicates and three biological replicates per each curve point.

Before starting cytotoxicity assays to characterize the chemosensitivity of cell lines to study drugs, different time of exposures and doses were tested in order to establish the most suitable working conditions. Lurbinectedin was tested exposing the cells at three different time points, 1 h, 24 h and 72 h. In all cases, cell viability analysis was performed 72 h after treatment administration. Regarding olaparib, exposure times were established at 72, 96 and 120 h, measuring absorbance directly after treatment exposure (Supplementary Fig. 10).

Based on the obtained results from the different tests performed with each drug and considering the subsequent administration of both drugs simultaneously, treatment conditions were fixed to 72 h of exposure followed by cell viability assay. Regarding lurbinectedin tests, cell viability results were comparable when exposing the cells during 24 and 72 h, while treating them during just 1 h was not enough to reach the IC50 value in the ranged concentrations. Due to olaparib’s molecular action inhibiting PARP, it was found that the higher time exposure, the lower IC50 values, changing proportionally. Hence, considering the concordance and suitability for applying 72 h of exposure times to both drugs, this time point was selected as the treatment scheme.

For MTT assay, cells were plated and after 24 h of incubation, treated with the drug (lurbinectedin/olaparib) either as a single agent or with both agents simultaneously. MTT reagent was added to the samples 72 h after treatment administration at a concentration of 0.5 mg/mL and incubated during 4 h. After MTT removal, propranolol was added and mixed for 15 min. Finally, the absorbance was measured at 570 nm.

For combination treatments (lurbinectedin + olaparib), cells were treated with increasing doses of lurbinectedin and fixed doses of olaparib, using the calculated IC30 and IC50.

Generated data were analyzed using the Compusyn software (ComboSyn Inv.) and GraphPad Prism Software (GraphPad Inc.). We used the Chou–Talalay combination index (CI) method to quantify synergistic interactions between the drugs tested^33^.

#### Blood collection and peripheral blood mononuclear cell isolation

Peripheral blood samples were collected at four different time points during the first cycle; before treatment administration and 4.5, 5.5 and 24 h after ending lurbinectedin infusion. Additional sample at the end of treatment visit was also collected*.*

For Peripheral Blood Mononucleated Cell (PBMC) isolation, a total of 8 mL of peripheral blood were collected in EDTA tubes and separated in Histopaque (SIGMA) gradient by centrifugation. The solution was centrifuged at 290*g* for 20 min at 24 °C. Then PBMC layer was carefully collected using a Pasteur pipette, and cells were washed twice in PBS (5 min/1300 rpm/4 °C). Cells were resuspended in saline solution and centrifuged 90 s at 13,000 rpm. Following this, supernatant was removed, and pelleted lymphocytes were stored at − 80 °C until their use for subsequent analyses.

#### Western blot analysis

PMBCs were lysed using 50–100 μL of lysis buffer (25 mM Tris–HCl pH 7.5, 1 mM EDTA pH 8,1 mM EDTA pH 8 and 1% SDS supplemented with protease and phosphatase inhibitors cocktail; leupeptine, pepstatine and Phenylmethylsulfonyl fluoride (PMSF)) depending on the characteristics of the sample. PMBCs were incubated 10 min at 95 °C, centrifuged at 16,000*g* for 10 min and maintained on ice. Once isolated, protein concentration was quantified by PierceTM BCA Protein assay Kit (ThermoFisher) following manufacturer’s protocol.

A total amount of 40 µg of protein per sample was separated on a 14% SDS-PAGE at 100 V and transferred into a nitrocellulose membrane during 3 h at 30 V (4 °C). Membranes were blocked for 1 h in 5% milk in Tris-buffered saline (TBS)-Tween, and then, incubated with primary antibodies in 5% non-fat milk overnight at 4 °C. The following day, they were washed four times with TBS-T for a total of 30 min. Finally, secondary antibodies were incubated 1 h at room temperature and washed four times with TBS-T.

Membranes were revealed using ECL Prime Western Blotting System (Sigma Aldrich) according to the manufacturer's instructions. To assess the presence of comparable amount of proteins in each lane (normalize the samples), membranes were stripped and incubated with β-actin (housekeeping protein).

Antibodies used were anti-RAD51 rabbit polyclonal antibody (PA5-27195, Thermofisher), dilution 1:1000, Anti-phospho-histone γH2AX mouse monoclonal antibody (Ser139, clone JLW301, MERCK) dilution 1:5000 and Anti-β-Actine antibody (Anti-beta Actin antibody (ab8227), abcam) dilution 1:3000. Secondary antibodies against mouse (1:3000, Dako, Denmark) and rabbit (1:3000, Dako, Denmark) were used.

#### Genotyping

DNA extraction was performed using 3 × 20 μm-thin sections of formalin-fixed and paraffin-embedded (FFPE) archived tumors and QIAmp DNA FFPE Tissue kit (Qiagen). The final concentration was measured spectrophotometrically using Nanodrop ND-1000 (Eppendorf). Additionally, concentration was determined fluorometrically with PicoGreen reagent using a quantifluor (Promega) instrument.

Libraries were prepared using the Agilent (Santa Clara, CA, USA) SureSelect-XT Target Enrichment Kit. Briefly, 200 ng of Extracted DNA was sheared on a Covaris M220 (Covaris) and fragmented to a size ranged between 150 and 200 bp. DNA integrity and fragment size were determined by a bioanalyzer, TapeStation 4200 (Agilent). Each library was then hybridized to a SureSelect custom panel (Agilent) according to the manufacturer’s protocol. The custom panel, designed to evaluate HRD status, included 35 genes involved in different DNA repair pathways; *BRCA1, BRCA2, BARD1, BRIP1, CHEK1, CHEK2, FAM175A, NBN, PALB2, ATM, MRE11A, RAD51B, RAD51C, RAD51D, RAD54L, FANCI, FANCM, FANCA, ERCC1, ERCC2, ERCC6, REQL, XRCC4, HELQ, SLX4, WRN, ATR, PTEN, CCNE1, EMSY, TP53, MLH1, MSH2, MSH6* and *PMS2*. Pooled library was sequenced on a HiSeq2500 (Illumina, San Diego, CA, USA), using a 2 × 150-bp protocol. For annotation, the following categories were considered pathogenic, likely pathogenic or variants of uncertain significance (VUS) with pathogenic prediction or variants with both in silico predictors, SIFT and Polyphen, predicted as pathogenic. Variants were filtered based on coverage and functional annotation. Minimum coverage established for a variant was 100X. Median coverage in filtered variants was 656 reads (range 154–2158 reads). Mutations were accepted with a frequency higher than 5%.

### Statistical analysis

Safety and efficacy population comprised all patients who received at least one dose of the study treatment. Continuous variables were presented with summary statistics and qualitative variables with the absolute and relative frequencies. In those cases where it was necessary to ensure the normality of the continuous parameters, log transformation (or other suitable transformation) was performed. Analysis of Variance (ANOVA) was used for exploratory group comparisons. If Gaussian distribution assumptions (Shapiro–Wilks test) were not met, equivalent nonparametric methods as the Kruskal–Wallis, were performed. Chi-squared or Fisher exact test were used for qualitative parameters. To explore the change in value of a variable, between baseline and week 12 or any other time point, a t-test for paired data or the Wilcoxon signed-rank test was performed, based on the distribution followed by the corresponding variable. All statistical analyses were performed using IBM SPSS Statistics 22 and R v.3.4.3. PK analysis was performed according to the NCA. PK parameters were tabulated, and selected parameters were graphically displayed per DL for olaparib and lurbinectedin. The dose-exposure relationships for Cmax and AUC for lurbinectedin and AUC 0–6 for olaparib will be evaluated and any potential PK interaction between olaparib and lurbinectedin were also explored. The population pharmacokinetics (PopPK) models for each drug was used to detect potential drug–drug interactions based on single agent PopPK models. The potential influence on selected PK parameters of selected demographic and clinical dichotomous variables (gender, laboratory test results above/below selected cutoff values, etc.) was evaluated by Student’s test Mann–Whitney’s U test as appropriate. For multinomial variables, analysis of variance was used. For selected continuous demographic and clinical variables, relationships with selected PK parameters were graphically explored and assessed using correlation and regression methods.

## Supplementary Information

Supplementary Information.
