# Supplementary material for: A phase I dose-finding, pharmacokinetics and genotyping study of olaparib and lurbinectedin in patients with advanced solid tumors
Source: Sci Rep. 2021 Feb 24;11:4433. doi: 10.1038/s41598-021-82671-w (PMC7904806; doi:10.1038/s41598-021-82671-w)

Manuscript Supplementary Material

Title:

A phase I dose-finding, pharmacokinetics and genotyping study of olaparib and lurbinectedin in patients with advanced solid tumors

Authors:

Andres Poveda[1], Ana Oaknin[2], Ignacio Romero[3], Angel Guerrero-Zotano[3], Lorena Fariñas-Madrid[2], Victor Rodriguez-Freixinos[4], Pedro Mallol[5], Raquel Lopez-Reig[6,7], Jose Antonio Lopez-Guerrero[6,7,8]

Affiliations

[1]Oncogynecologic Department, Initia Oncology, Hospital Quironsalud Valencia, Valencia

[2]Medical Oncology Department. Vall d´Hebron University Hospital. Vall d´Hebron Institute of Oncology (VHIO). Barcelona, Spain

[3]Department of Medical Oncology, Fundación Instituto Valenciano de Oncología (IVO), Valencia, Spain.

[4]Department of Medical Oncology and Hematology, Odette Cancer Centre, Sunnybrook Health Sciences Centre, Toronto, Canada

[5]Clinical Trials Department, FINCIVO (Fundación de Investigación Clínica del IVO), Valencia, Spain.

[6] Laboratory of Molecular Biology, Fundación Instituto Valenciano de Oncología, Valencia, Spain

[7] IVO-CIPF Joint Research Unit of Cancer, Príncipe Felipe Research Center (CIPF), Valencia, Spain

[8]Department of Pathology, School of Medicine, Catholic University of Valencia ‘San Vicente Martir’, Valencia, Spain

Corresponding author:

Andres Poveda, MD
[1] Oncogynecologic Department, Initia Oncology, Hospital Quironsalud Avda Blasco Ibañez, 14, 46 010-Valencia, Spain. E-mail: apoveda@initiaoncologia.com

Table of Contents

Definition of Dose Limiting Toxicity (DLT). 3
Supplementary Table 1. Adverse events observed in ≥10% of all patients 4
Supplementary Table 2. Pharmacokinetic parameters by lurbinectedin dose 6
Supplementary Table 3. Pharmacokinetic parameters by olaparib dose 7
Supplementary Table 4. Histology, HDR status and best response by patient. 8
Supplementary Table 5. Sampling schedule for the determination of lurbinectedin and olaparib in Cycle 1. 9
Supplementary Table 6. Cell lines information. 10
Supplementary Figure 1. Mean Lurbinectedin (PM01183) Clearance by dose level. 11
Supplementary Figure 2. Mean Lurbinectedin Clearance by Olaparib dose level. 11
Supplementary Figure 3. Mean olaparib clearance by olaparib dose level. 12
Supplementary Figure 4. Kaplan Meier model – Time to Radiological Progression curve-global population 12
Supplementary Figure 5. Time to radiological progression by dose level 13
Supplementary Figure 6. Dose-response curve for Lurbinectedin (PM001183). 14
Supplementary Figure 7: Dose-response curve for Olaparib (OLA). 15
Supplementary Figure 8: Distribution of IC50 values between cell lines classified based on HR status. 16
Supplementary Figure 9: Synergistic interaction between Lurbinectedin and Olaparib. 16
Supplementary Figure 10: Evaluated experimental parameters for cytotoxicity assays 17

Definition of Dose Limiting Toxicity (DLT).

Dose-limiting toxicity (DLT) was defined according to the following criteria:

1. Hematological and blood chemistry treatment-related AEs:
   a. Any grade 4 neutropenia (ANC < 0.5 $x10^9/L$) lasting more than 5 days.
   b. Any grade 4 neutropenia with concomitant fever (i.e., body temperature ≥ 38.5°C). Fever should not be disease-related.
   c. Any grade 4 neutropenia with sepsis or other severe infection. o Grade ≥γ febrile neutropenia as defined in the CTCAE v 4.0 o Grade 4 thrombocytopenia or grade 3 complicated by hemorrhage or lasting more than 5 days. o Grade 4 anemia.
   d. Grade 3-4 hypoalbuminemia.
   e. Grade 3-4 AST/ALT elevation lasting ≥7 days (exception may be made for elevations in the presence of liver metastases and without evidence of other hepatic injury, if agreed by investigator).
2. Any other grade 3-4 non-hematological AEs related to the study treatment, except for the following:
   a. Grade 3 fatigue, nausea and vomiting, diarrhea, unless appropriate prophylactic or therapeutic measures have been administered.
   b. Non-clinically relevant biochemical abnormalities [i.e., isolated increase of gamma glutamyltransferase (GGT)].
   c. Hypersensitivity reactions.
3. Grade 2 cardiac or neurological toxicity.
4. Inability to tolerate the cycle of therapy due to toxicity.
5. Any toxicity, which in the judgement of the Sponsor and investigator is viewed as DLT.

In order to define DLT, patients should not be prophylactically prescribed growth factor support, during cycle 1 of therapy.

If a patient experiences grade 2 or greater nausea and/or vomiting or diarrhea, medical intervention should occur, including prophylactic treatment for subsequent doses as indicated. If in a given dose level, patients experience grade 2 or greater nausea or vomiting (e.g. at least one patient with >=G2 in one dose level), antiemetics will be highly recommended for following patients, including 1st cycle in the following dose levels.

In case a grade 3-4 hematologic toxicity appears, blood tests will be repeated every 24 hours until grade ≤1.

Supplementary Table 1. Adverse events observed in ≥10% of all patients

Adverse event	DL1 n = 3		DL2 n = 3		DL3 n = 4		DL4 n = 6		DL5 n = 4		Total n = 20	
Anemia, n (%)												
Grade 1-2	1	(33.3)					1	(16.7)	1	(25)	3	(15)
Grade 3-4					2	(50)					2	(10)
Neutropenia, n (%)												
Grade 1-2							1	(16.7)	1	(25)	2	(10)
Grade 3-4					1	(25)			2	(50)	3	(15)
Thrombocytopenia, n (%)												
Grade 1-2					1	(25)			1	(25)	2	(10)
Grade 3-4												
Abdominal distension, n (%)												
Grade 1-2			1	(33.3)	2	(50)					3	(15)
Grade 3-4												
Abdominal pain, n (%)												
Grade 1-2			2	(66.7)	2	(50)	2	(33.3)	2	(50)	8	(40)
Grade 3-4												
Constipation, n (%)												
Grade 1-2	1	(33.3)	1	(33.3)	2	(50)	1	(16.7)	4	(100)	9	(45)
Grade 3-4												
Diarrhoea, n (%)												
Grade 1-2					4	(100)			1	(25)	5	(25)
Grade 3-4												
Nausea, n (%)												
Grade 1-2	3	(100)	1	(33.3)	1	(25)	2	(33.3)	4	(100)	11	(55)
Grade 3-4												
Vomiting, n (%)												
Grade 1-2	2	(66.7)			3	(75)	1	(16.7)	4	(100)	10	(50)
Grade 3-4												
Stomatitis, n (%)					2	(50)					2	(10)
Grade 1-2												
Grade 3-4												
Asthenia, n (%)												
Grade 1-2	2	(66.7)	2	(66.7)	2	(50)	1	(16.7)	1	(25)	8	(40)
Grade 3-4					1	(25)			2	(50)	3	(15)
Pyrexia, n (%)												
Grade 1-2	1	(33.3)			1	(25)	1	(16.7)	2	(50)	5	(25)
Grade 3-4												
Urinary tract infections, n (%)												
Grade 1-2							2	(33.3)			2	(10)
Grade 3-4												
Anorexia, n (%)												
Grade 1-2	1	(33.3)			2	(50)			3	(75)	6	(30)
Grade 3-4												
Hypomagnesemia, n (%)												
Grade 1-2	1	(33.3)			1	(25)			1	(25)	3	(15)
Grade 3-4												
Arthralgia, n (%)												
Grade 1-2	2	(66.7)			1	(25)	1	(16.7)			4	(20)
Grade 3-4												

Back pain, n (%)												
Grade 1-2	2	(66.7)	1	(33.3)							3	(15)
Grade 3-4												
Myalgia, n (%)												
Grade 1-2	1	(33.3)			1	(25)	1	(16.7)			3	(15)
Grade 3-4												
Dysgeusia, n (%)												
Grade 1-2	1	(33.3)	2	(66.7)	1	(25)	2	(33.3)	3	(75)	9	(45)
Grade 3-4												
Headache, n (%)												
Grade 1-2							1	(16.7)	1	(25)	2	(10)
Grade 3-4												
Insomnia, n (%)												
Grade 1-2	1	(33.3)	1	(33.3)							2	(10)
Grade 3-4												
Cough, n (%)												
Grade 1-2			1	(33.3)	2	(50)					3	(15)
Grade 3-4												

Supplementary Table 2. Pharmacokinetic parameters by lurbinectedin dose

PM01183 DL (mg/m²)		Cmax (ug/L)	AUC (h*ug/L)	CL (L/h)	HL (h)	Vss (L)	Vz (L)	Tlast (h)
1	N	5	5	5	5	5	5	5
	Mean	47.5	188.0	14.4	36.9	461.2	653.2	122.7
	SD	19.4	94.1	13.3	16.3	279.4	422.9	48.4
	Min	25.9	54.0	6.0	19.9	127.4	173.4	69.6
	Median	47.9	246.4	6.7	35.5	440.1	587.3	141.3
	Max	67.5	263.4	37.0	60.6	752.9	1286.6	165.8
	CV%	40.8	50.1	92.2	44.3	60.6	64.7	39.5
1.5	N	9	9	9	9	9	9	9
	Mean	60.9	284.4	11.3	56.1	566.4	858.7	140.6
	SD	25.5	151.9	4.1	36.0	370.3	552.5	47.4
	Min	28.3	143.3	4.3	14.4	199.0	282.0	46.2
	Median	53.4	245.8	11.6	52.7	385.8	699.7	167.5
	Max	101.0	609.0	16.1	129.8	1175.9	1839.0	168.1
	CV%	41.9	53.4	35.9	64.2	65.4	64.3	33.7
2	N	4	4	4	4	4	4	4
	Mean	57.7	364.0	11.0	58.6	498.6	671.8	142.4
	SD	13.3	225.8	5.7	50.8	187.9	202.6	47.7
	Min	37.9	178.4	4.6	21.0	285.0	448.4	71.0
	Median	63.4	293.0	10.5	40.2	487.4	676.1	165.3
	Max	66.1	691.5	18.5	132.8	734.5	886.8	168.0
	CV%	23.0	62.0	51.8	86.8	37.7	30.1	33.5

C_{max}: Maximum plasma concentration, **AUC** Area under the plasma concentration-time curve from time zero to infinite, **HL**: Terminal half-life, **CL**: Total body clearance, **V_{ss}** : volume of distribution at steady-state, **V_z**: Volume of distribution based on terminal phase, **N**: number el patients/cycles, **Mean**: arithmetic mean, **SD**: standard deviation, **CV%**: Coefficient of variation.

Supplementary Table 3. Pharmacokinetic parameters by olaparib dose

Olaparib DL (mg BID)		Cmax (h*ug/L)	AUC (L/h)	CL (h)	HL (L)	Vss (L)	Vz (h)	Tlast (h)
100	N	2	2	2	2	2	2	2
	Mean	66.7	254.9	6.3	32.1	191.1	294.1	118.9
	SD	1.1	12.0	0.3	17.2	90.0	170.7	66.3
	Min	65.9	246.4	6.0	19.9	127.4	173.4	72.0
	Median	66.7	254.9	6.3	32.1	191.1	294.1	118.9
	Max	67.5	263.4	6.5	44.3	254.8	414.8	165.8
	CV%	1.7	4.7	5.2	53.7	47.1	58.0	55.8
150	N	3	3	3	3	3	3	3
	Mean	34.7	143.4	19.8	40.0	641.3	892.6	125.3
	SD	11.7	100.9	15.6	18.7	174.6	358.0	49.7
	Min	25.9	54.0	6.7	24.1	440.1	587.3	69.6
	Median	30.2	123.4	15.7	35.5	731.0	804.1	141.3
	Max	47.9	252.9	37.0	60.6	752.9	1286.6	165.0
	CV%	33.6	70.4	78.5	46.6	27.2	40.1	39.6
200	N	4	4	4	4	4	4	4
	Mean	69.8	346.8	9.1	80.2	751.1	1076.2	162.3
	SD	35.0	178.5	3.3	38.5	465.1	664.9	11.1
	Min	28.3	217.6	4.3	41.9	313.1	369.3	145.7
	Median	74.9	280.2	10.3	74.6	757.7	1048.3	167.7
	Max	101.0	609.0	11.6	129.8	1175.9	1839.0	168.0
	CV%	50.1	51.5	36.1	48.0	61.9	61.8	6.8
250	N	9	9	9	9	9	9	9
	Mean	55.5	292.1	12.2	46.5	454.2	678.9	131.8
	SD	13.9	177.2	4.6	36.3	200.7	335.2	52.3
	Min	37.3	143.3	4.6	14.4	199.0	282.0	46.2
	Median	59.1	245.8	12.2	31.7	440.8	560.1	162.9
	Max	77.8	691.5	18.5	132.8	734.5	1188.2	168.1
	CV%	25.0	60.7	38.2	78.1	44.2	49.4	39.7

C_{max}: Maximum plasma concentration, **AUC** Area under the plasma concentration-time curve from time zero to infinite, **HL**: Terminal half-life, **CL**: Total body clearance, **V_{ss}** : volume of distribution at steady-state, **V_z**: Volume of distribution based on terminal phase, **N**: number el patients/cycles, **Mean**: arithmetic mean, **SD**: standard deviation, **CV%**: Coefficient of variation.

Supplementary Table 4. Histology, HDR status and best response by patient.

Patient ID	Histology	HRD status	Dose level	Best response
**CE1**	Endometrium	nonHRD	DL3	SD
**CE2**	Endometrium	nonHRD	DL3	PD
**CE3**	Endometrium	Unknown	DL5	PD
**CE4**	Endometrium	Unknown	DL5	SD
**CE5**	Endometrium	Unknown	DL4	SD
**CO1**	Ovary	HRD	DL1	SD
**CO2**	Ovary	HRD	DL5	SD
**CO3**	Ovary	HRD	DL2	PD
**CO4**	Ovary	HRD	DL4	SD
**CO5**	Ovary	HRD	DL1	SD
**CO6**	Ovary	HRD	DL1	PD
**C07**	Ovary	HRD	DL4	SD
**CO8**	Ovary	nonHRD	DL4	PD
**CO9**	Ovary	nonHRD	DL3	SD
**CO10**	Ovary	nonHRD	DL3	SD
**CO11**	Ovary	nonHRD	DL2	PD
**CO12**	Ovary	Unknown	DL4	PD
**CO13**	Ovary	Unknown	DL2	PD
**CO14**	Ovary	Unknown	DL5	SD
**CO15**	Ovary	Unknown	DL4	SD

CE, endometrial cancer; CO, ovarian cancer; DL, dose-level; HRD, homologous recombination deficiency; PD, progression disease; SD, stable disease.

Supplementary Table 5. Sampling schedule for the determination of lurbinectedin and olaparib in Cycle 1.

Sample number	Day	Time relative to the start of the lurbinectedin infusion	Sampling times for lurbinectedin	Sampling times for olaparib	PK window
#1	1	0 min	Pre-infusion	Pre-dose	-10 min to SOI
#2	1	0.5 h	30 min before EOI	30 min after 1st administration	±2 min
#3	1	0.92 h	5 min before EOI	55 min after 1st administration	±2 min
#4	1	1.5 h	30 min after EOI	1.5 h after 1st administration	±5 min
#5	1	3 h	2 h after EOI	3 h after 1st administration	±10 min
#6	1	4.5 h	3.5 h after EOI	4.5 h after 1st administration	±10 min
#7	1	6 h	5 h after EOI	6 h after 1st administration	±10 min
#8 □□	2	24 h	23 h after EOI	24 h after 1st administration (immediately before 3rd olaparib administration)	±2 h
#9 †	4	72 h	71 h after EOI	72 h after 1st administration (immediately before 7th olaparib administration)	+24 / -2 h
#10	8	168 h	167 h after EOI	168 h after 1st administration	±24 h

Supplementary Table 6. Cell lines information.

Cell Line	Organ	Tissue	Site	Histology	Genotype	HRD status
A2780	Ovary	Tumor tissue sample	Primary tumor	Ovarian endometrioid adenocarcinoma	ATM (p.P604S, 40%)<br>CHEK2 (p.Y372_373Tdel, 38%)<br>PTEN (p.K128_R130del, 100%)	HRD
A2780CIS	Ovary	Tumor tissue sample	Primary tumor	Ovarian endometrioid adenocarcinoma<br>Induced Cisplatin resistance	ATM (p.P604S, 40%)<br>CHEK2 (P.H194R, 43%)<br>PTEN (p.K128_R130del, 100%)<br>MSH6 (p.T1085_1086Pins, 33%)<br>ATR (p.I744_745Ydel, 11%)<br>NBN (p.N209Y, 24%)<br>TP53 (p.D7V, 36%)	HRD
PEO6	Ovary	Ascitis	Metastatic site	Ovarian cystadenocarcinoma<br>Hereditary breast and ovarian cancer syndrome	TP53 (p.G144D, 100%)<br>BRCA2 (p.Y1655, 100%) reversion	Primary HRD
TOV112	Ovary	NA	NA	Ovarian endometrioid adenocarcinoma	TP53 (p.R175H, 100%)	nonHRD
OVCAR-3	Ovary	NA	NA	High grade ovarian serous adenocarcinoma	TP53 (p.E248Q, 100%)<br>CCNE1 amp	nonHRD
SKOV-3	Ovary	Ascitis	NA	Ovarian serous cystadenocarcinoma	TP53 (p.S89_90Pdel, 100%)<br>MSH6 (p.S1329P, 33%)<br>EMSY (p.K1086_1087Ndel, 30%)	nonHRD

Supplementary Figure 1. Mean Lurbinectedin (PM01183) Clearance by dose level.

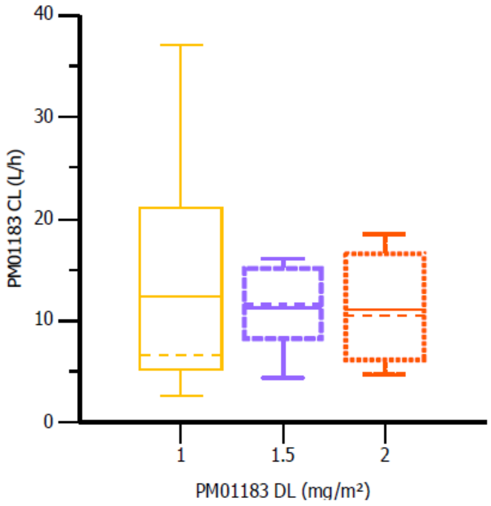

Supplementary Figure 2. Mean Lurbinectedin Clearance by Olaparib dose level.

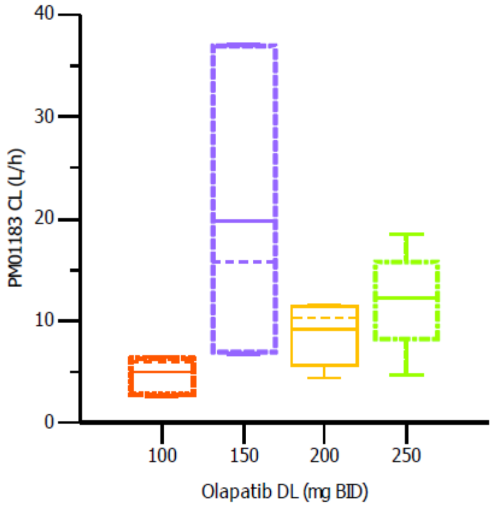

Supplementary Figure 3. Mean olaparib clearance by olaparib dose level.

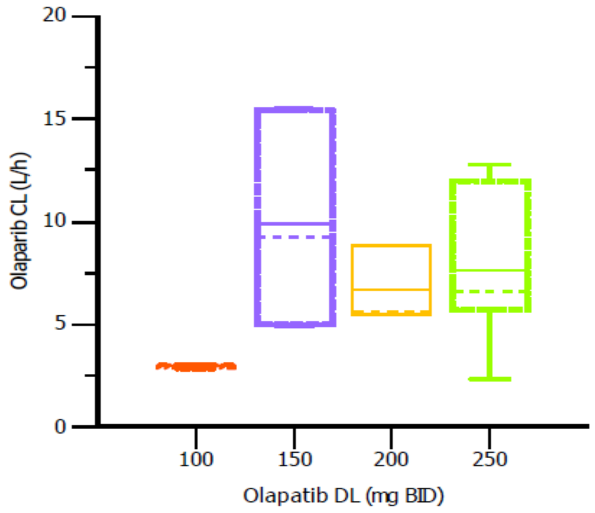

Supplementary Figure 4. Kaplan Meier model – Time to Radiological Progression curve-global population

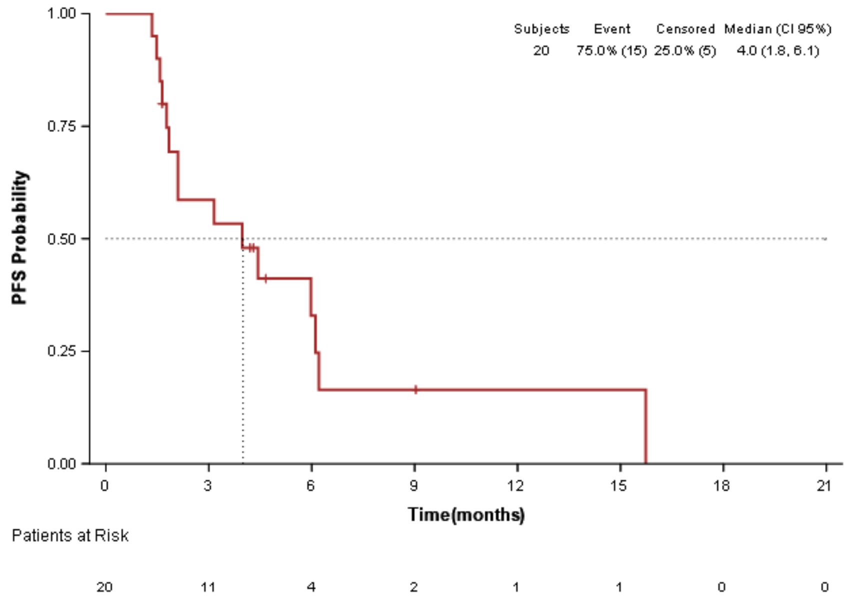

Supplementary Figure 5. Time to radiological progression by dose level

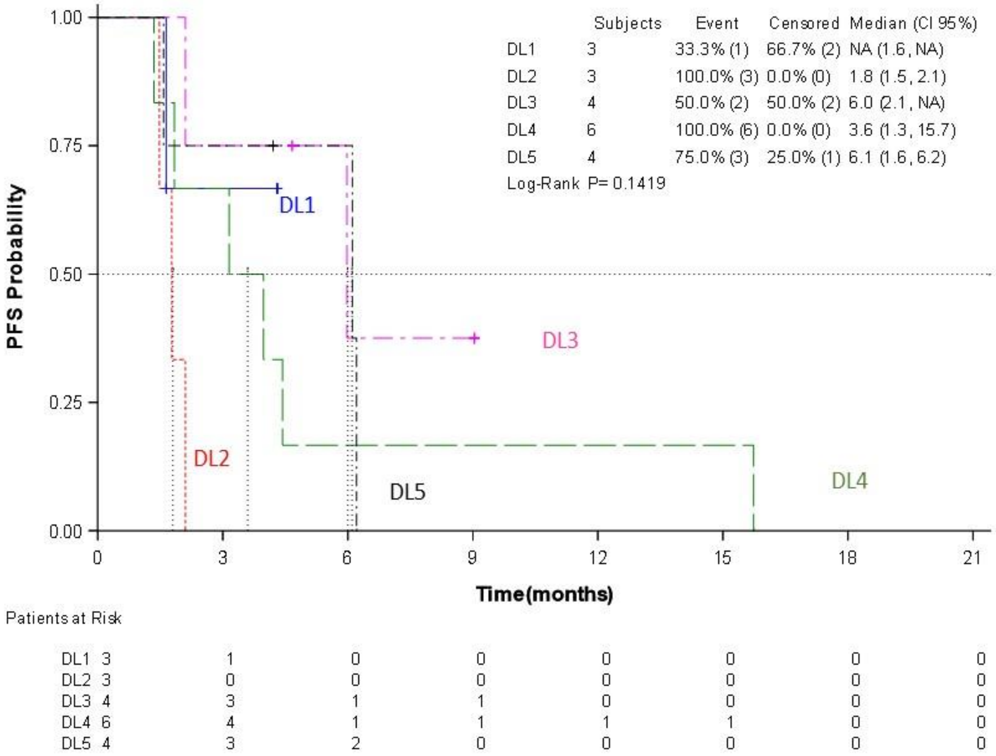

Supplementary Figure 6. Dose-response curve for Lurbinectedin (PM001183). Cell line showing the lowest IC50 was A2780 (0.46 μM). , Due to OVCAR3 lower duplication rate compared with the other cell lines, it was complicated to establish IC50 values following the same experimental approach.

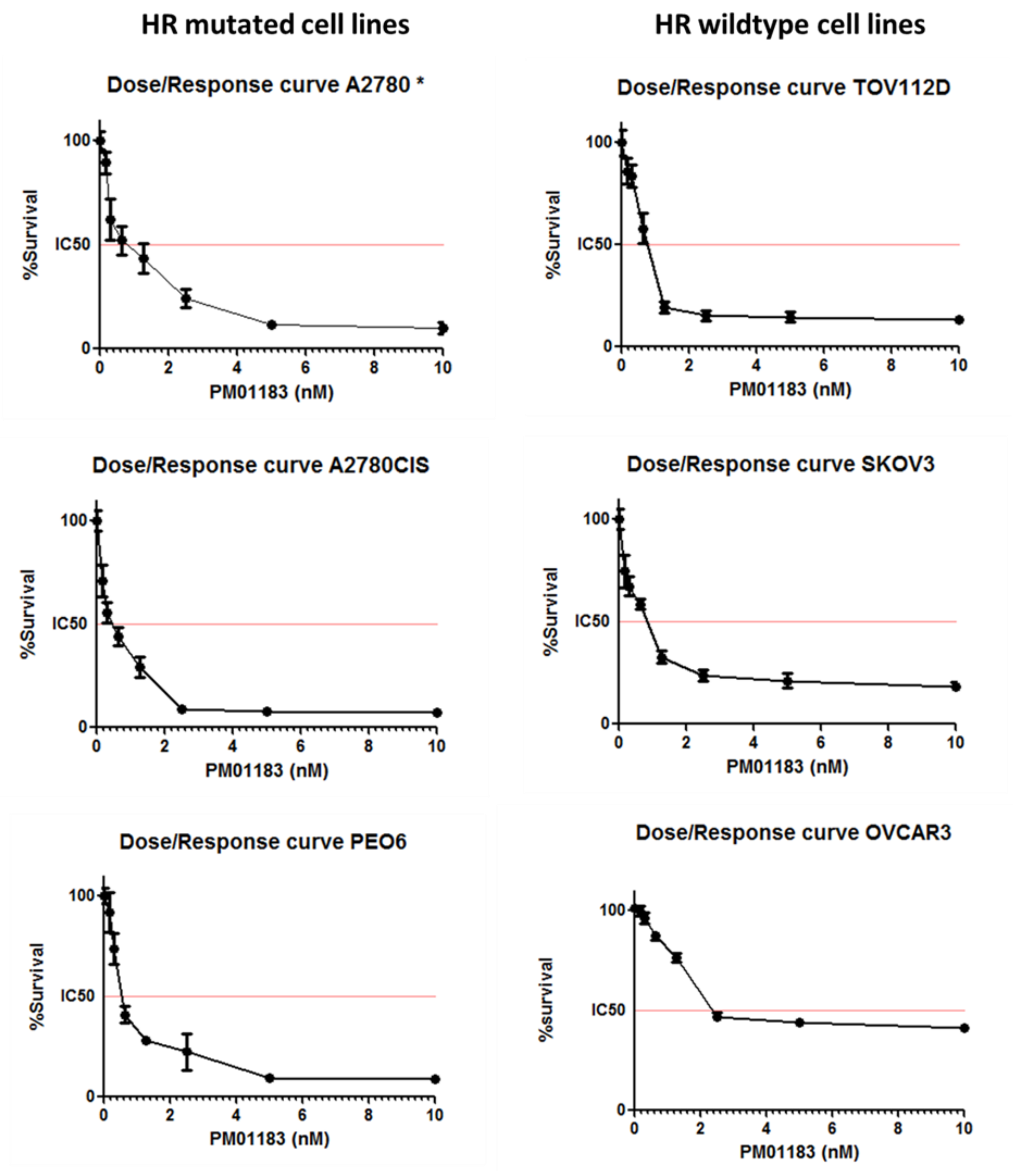

Supplementary Figure 7: Dose-response curve for Olaparib (OLA). Cell line showing the lowest IC50 A2780 CIS (2.9 µM).

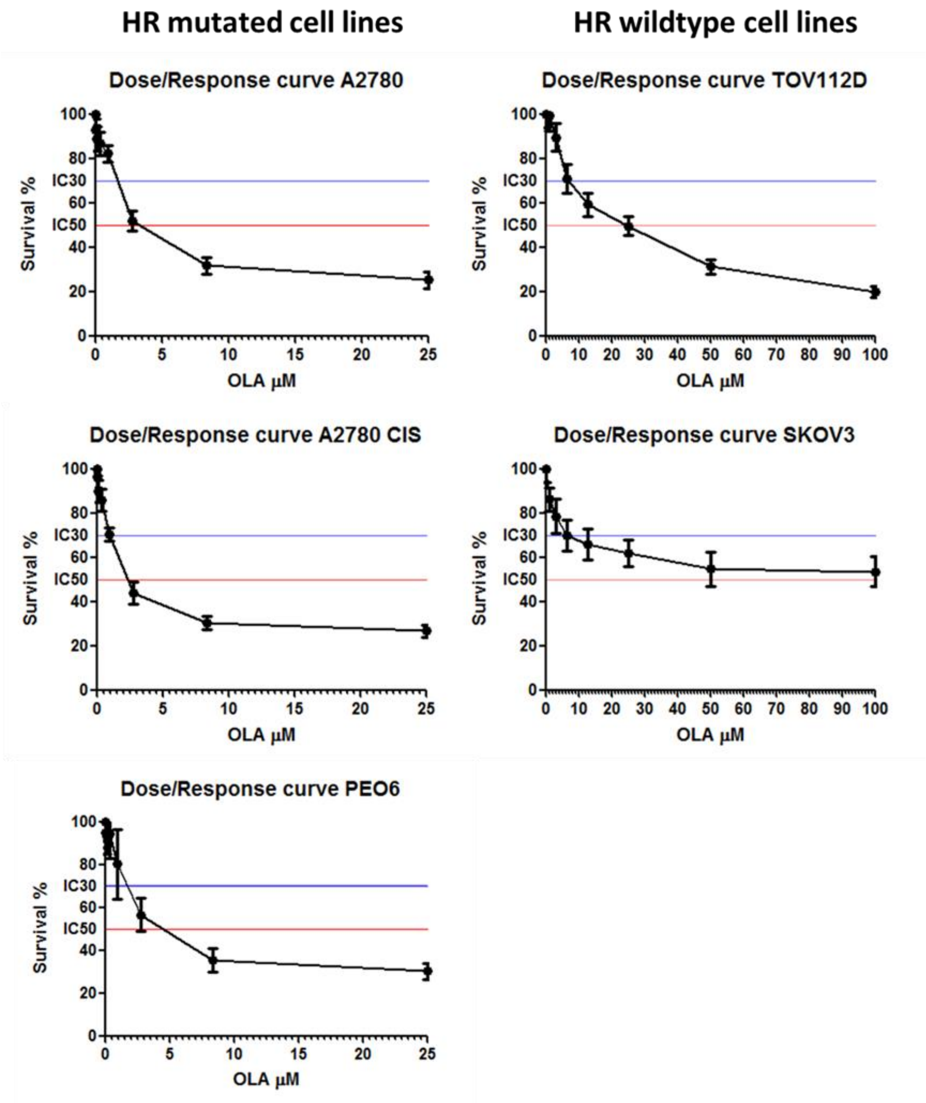

Supplementary Figure 8: Distribution of IC50 values between cell lines classified based on HR status.

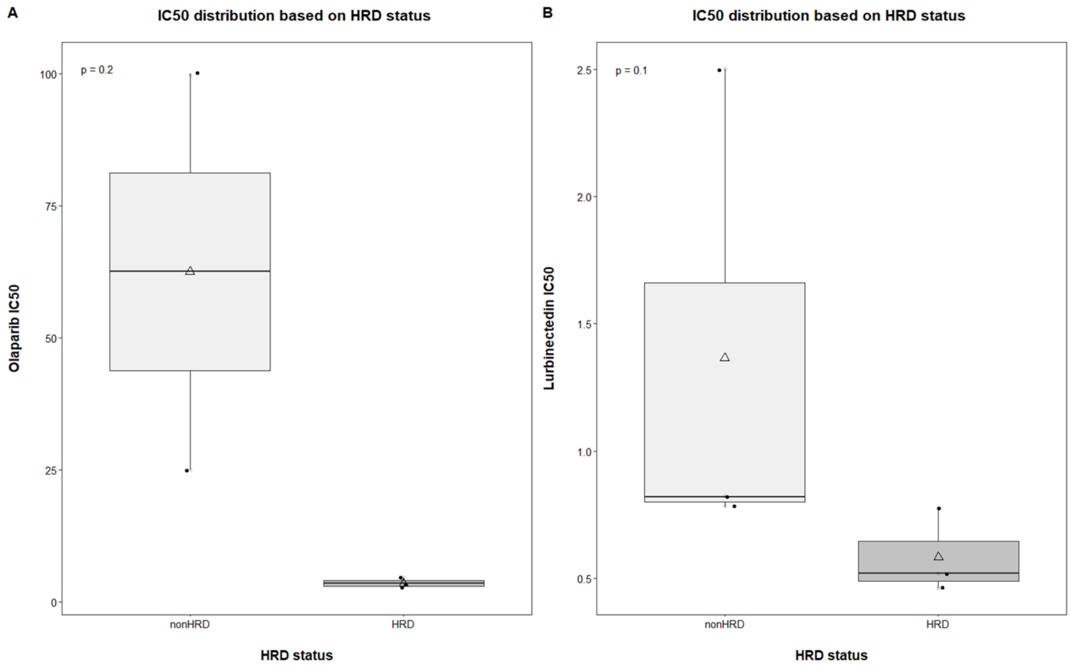

Supplementary Figure 9: Synergistic interaction between Lurbinectedin (PM01183) and Olaparib. According to Chou (2006), strong synergistic interactions occur when combination indexes (CI) are below 0.3. Hence, a synergistic interaction was observed in the lurbinectedin-Olaparib combinations across all tested dose levels.

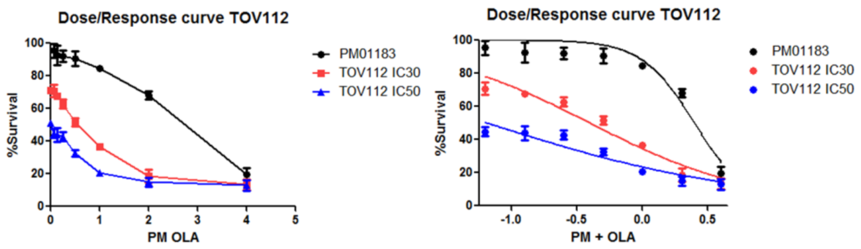

PM	OLA IC50	Effect	CI
0.0625	25.0	0.55312	0.33800
0.125	25.0	0.56077	0.33152
0.25	25.0	0.57259	0.32911
0.5	25.0	0.67439	0.17454
1.0	25.0	0.79387	0.10002
2.0	25.0	0.85096	0.10816

Supplementary Figure 10: Evaluated experimental parameters for cytotoxicity assays

A) Different tested conditions to Lurbinectedin (PM01183) cytotoxicity assays. B) Different tested conditions for PARP inhibitors cytotoxicity assays

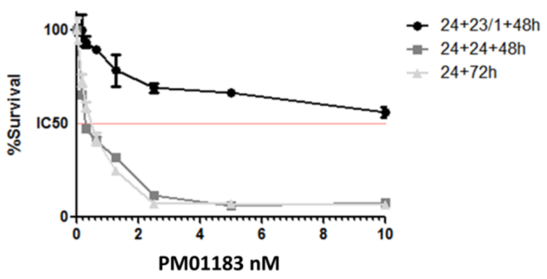

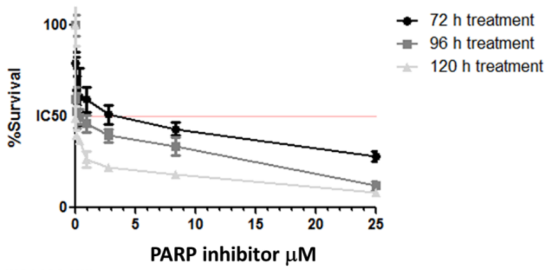

Supplement: Supplementary file 1 — Supplementary Information. [file 41598_2021_82671_MOESM1_ESM.pdf]
